# High-dose treatment with ADXS11-001, a listeria monocytogenes (Lm)-listeriolysin O (LLO) immunotherapy, in women with cervical cancer

**DOI:** 10.1186/2051-1426-3-S2-P151

**Published:** 2015-11-04

**Authors:** Sharad Ghamande, David Mauro, Cheryl Price, Donna Wheatley, John Janik, Samir N Khleif

**Affiliations:** 1 GRU Cancer Center, Georgia Regents University, Augusta, GA, USA; 2 Advaxis, Inc., Princeton, NJ, USA

## Introduction

Survival of patients with persistent and recurrent cervical cancer is dismal, and improvement remains a large unmet need. ADXS11-001 is a live, attenuated, bioengineered *Lm*-LLO immunotherapy for the treatment of human papillomavirus (HPV)-associated cancers such as cervical cancer. ADXS11-001 secretes an HPV-E7 tumor antigen as a truncated LLO-E7 fusion protein that stimulates antitumor immunity through T cells, while reducing immune tolerance via T-regulatory and myeloid-derived suppressor cells. ADXS11-001 has been shown to be safe and effective in women with recurrent/refractory cervical cancer. This Phase I study evaluates whether a higher ADXS11-001 dose than that currently used in Phase II trials is safe and well tolerated.

## Methods

This is a Phase I, dose-escalation, open-label study (NCT02164461) enrolling women aged ≥18 years with persistent, metastatic, or recurrent squamous/adenocarcinoma of the cervix and documented disease progression (not amenable to surgery/standard radiotherapy). Additional eligibility criteria include: measurable and/or evaluable disease per Response Evaluation Criteria in Solid Tumors (RECIST v1.1); Eastern Cooperative Oncology Group (ECOG) performance status of 0–1; and ≤2 prior treatments for metastatic disease. The primary endpoint is the safety and tolerability of ADXS11-001; secondary endpoints include evaluating tumor response and progression-free survival, and assessing correlative immunologic studies. Patients will receive ADXS11-001 every 3 weeks during a 12-week treatment cycle. Dose escalation is performed using the 3+3 design in 2 doses: 5x10^9^ colony-forming units (CFU; Dose Level 1) and 1x10^10^ CFU (Dose Level 2). The recommended Phase II dose will be selected based on an observed dose-limiting toxicity (DLT) rate of < 33%. Efficacy is assessed using RECIST v1.1 and immune-related RECIST. Blood samples will be collected in cycle 1 only and used for immune monitoring and cytokine/chemokine analysis.

## Results

Enrollment into Dose Level 1 is complete (n=6). Initially 3 patients were enrolled into the first dose cohort; 1 patient experienced grade 3 hypotension as a DLT, resulting in 3 additional patients being enrolled. The mean age is 51.3 years, 66.7% (n=4) had ECOG 0 at baseline, and 83.3% (n=5) patients have squamous histology. All patients received prior cisplatin-based concurrent chemoradiation, plus a median of 1.5 (range 0–5) lines of systemic chemotherapy. A total of 16 doses of ADXS11-001 have been safely administered; accrual for Dose Level 2 is starting. Updated data on the determination of the maximum tolerated dose and efficacy of ADXS11-001 will be presented.

## Trial registration

ClinicalTrials.gov identifier NCT02164461.

